# Changes in Electromagnetic Activity Following Osteopathic Manipulative Treatment: A Novel Assessment Using Induction Sensors

**DOI:** 10.7759/cureus.88819

**Published:** 2025-07-26

**Authors:** Paras Savla, James Brazdzionis, Maxwell Marino, Imran Siddiqi, Raed Sweiss, Christopher King, Dan E Miulli

**Affiliations:** 1 Neurosurgery, Riverside University Health System Medical Center, Moreno Valley, USA; 2 Neurosurgery, Aurora Medical Center, Summit, USA; 3 Neurosurgery, Arrowhead Regional Medical Center, Colton, USA

**Keywords:** electromagnetic field stimulation, electromagnetic frequencies therapy, electromagnetic frequency, osteopathic manipulative medicine, osteopathic treatment

## Abstract

Background

Electromagnetic induction sensors have been utilized to measure neuronal signaling using a novel Mu-metal shielded helmet constructed with electromagnetic field (EMF) channels in animal studies as well as in patient populations. These sensors have discerned healthy controls from patients with neural pathologies. Osteopathic manipulative treatment (OMT) in the form of suboccipital tension release is thought to modulate neuronal function and cortical pathways. This study aimed to evaluate whether these EMF changes could be identified post-treatment.

Methods

A pilot study was conducted with 35 subjects who had EMF measurements obtained prior to and after OMT treatment with compression of the fourth ventricle (CV4). EMF measurements were obtained using induction sensors and a shielded helmet and then transformed using a Fast Fourier Transform algorithm. The transformed data were subsequently post-processed to evaluate changes in neural firing patterns.

Results

A total of 35 patients consented to this study and underwent CV4 after baseline assessment of EMF signals. Post-OMT measurements were obtained immediately after treatment. It was observed that there was increased synchronicity in the brain waves after treatment, as evidenced by more pronounced peaks and valleys and waves being closer together in amplitude. Additionally, a transition from negative to positive waveforms can be observed at lower frequencies following effective treatment, accompanied by positive waves in the mid frequencies.

Conclusion

Novel induction sensors appeared efficacious in evaluating neural circuitry related to OMT-related changes post-treatment. OMT appeared to induce changes in slope variability, which may be linked to increased synchronicity of the brain waves, in addition to causing a reversal of negative valleys to peaks. These overall effects will need to be evaluated and confirmed in future studies.

## Introduction

Electromagnetic field (EMF) induction sensors are an evolving technology that allows for non-invasive measurement of electromagnetic activity generated by neurons participating in cortical circuits [1-5]. These sensors have been previously utilized to detect differences in EMF patterns between patients with stroke or traumatic brain injury (TBI) when compared to healthy controls [6]. To conduct measurements, these induction sensors rely on shielding and are integrated into a novel, lightweight, and portable helmet apparatus that shields the brain and sensors from external EMF noise, enabling real-time, non-invasive recording of EMF waves produced by cortical circuits [3]. Studies have demonstrated the ability of this helmet-sensor configuration to discern changes in neural EMF associated with emotional thoughts, movement planning, and motor activities [1-5]. Studies have also identified the capabilities of these sensors to detect pathologic effects correlated with histological changes in vivo in a swine model [7-9].

Osteopathic manipulative treatment (OMT) incorporates various hands-on techniques, such as stretching, mobilization, and soft tissue manipulation. Techniques, such as compression of the fourth ventricle (CV4), an OMT technique applied to the cervical musculature and base of the skull, have been proposed to affect autonomic, sympathetic, and parasympathetic tone [10]. However, there is limited research on the neurophysiological effects of OMT using quantitative measures. Furthermore, there is the supposition that OMT may alter glymphatic flow and, secondarily, optimize the neuronal environment by promoting the clearance of abnormal proteins and reducing elevated intracranial pressures [11].

Based on the autonomic and neuromodulatory effects reported with OMT, we hypothesized that OMT may alter neuronal activity. EMF changes picked up by the helmet-sensor technology (helmet) following OMT may provide novel neurophysiological evidence of these potential effects. Quantifying EMF patterns associated with OMT represents an innovative approach, building upon prior research that demonstrates the utility of this helmet in detecting differences in cortical EMF signaling through multiple pathways to optimize the neural environment [2,3,5].

This study aimed to determine whether the EMF helmet could identify changes in EMF activity via waveform morphology, synchronization, and slope variability following a session of suboccipital release OMT. Detecting OMT-induced neural effects would further validate the sensitivity of this technology in measuring real-time modifications of cortical circuit dynamics non-invasively and identify neurophysiological changes associated with OMT.

## Materials and methods

This study was a pilot study designed to evaluate EMF changes before and after OMT. The study included 35 adult participants (10 male and 25 female) scheduled to receive OMT from a single licensed osteopathic physician on a single day. The inclusion criterion was a patient age of 18 years or older. The exclusion criteria were prior neck surgery or absolute contraindications to OMT. A target sample size of 35 participants was set based on feasibility and budget considerations. The study procedures were approved by the Arrowhead Regional Medical Center Institutional Review Board prior to initiation (approval no. ARMC IRB 21-05).

Demographic information, including age, gender, and race, was collected. Relevant medical history and current medications were also recorded. The intervention consisted of a single session of suboccipital release OMT performed by a Doctor of Osteopathic Medicine (DO). This technique involves gentle pressure and mobilization applied to the neck muscles and cranial-cervical junction to achieve relaxation and hypothesized autonomic effects [12-16]. The physician performing the treatment places both hands with thumbs applying pressure to the junction. The physician feels for a release in the tissue tension, upon which the treatment is deemed complete. The OMT session lasted approximately 5-10 minutes based on the physician's clinical judgment. The time varied based on the length of time to feel this release, as a standard amount of treatment time would not be specific to each patient.

The primary outcome was EMF activity measured non-invasively using an EMF helmet. This helmet was constructed using dual-layer Mu-metal (MuMETAL®, Magnetic Shield Corporation, Bensenville, IL), interlaced copper mesh, and an air gap constructed with EMF channels, as in previous studies [1-5]. The induction sensors (BS-1000; Quasar Federal Systems, San Diego, California) detect electromagnetic waves generated by cortical neural circuits without direct contact with the scalp. Participants wore the EMF helmet in a seated position for 30 seconds before and immediately after the OMT session to obtain comparative EMF recordings. Measurements were also taken after two minutes of meditation for comparison to OMT. The lightweight helmet was suspended over the head to secure the positioning and avoid contact with the patient's scalp [2,3]. A chair was placed under the helmet for the patient to sit on. Data were acquired at a rate of 5,000 samples per second for each of the 16 sensors.

The induction sensors were positioned bilaterally on the helmet over all regions of the brain to detect EMF patterns generated specifically by neurons within these areas. A total of 16 induction sensors were positioned on each side equidistant from the midline. The sensors were numbered from 801 to 821, skipping 801, 804, 810, 814, and 818 due to the production numbering system. All sensors are built the same way. Measurement was conducted as previously published [1-7]. In addition, measurements at rest were taken from 9 inches away to narrow the measurement cone, allowing for more specific analysis.

The raw EMF data were collected using previously established techniques and analyzed using a Fast Fourier Transform algorithm in Igor Pro 8 software (Wavemetrics Inc., Lake Oswego, OR) to examine the frequency domain. Raw data were collected and binned into 20-second bins using previously reported techniques [1-7]. Quantitative and qualitative assessments of the waveform morphology were evaluated by the authors, focusing on identifying changes in the location and amplitude of peaks, valleys, and slope variations when comparing pre- and post-OMT measurements. AI software (ChatGPT 4o, OpenAI, DE, USA) was used to confirm observations seen in the pre- and post-OMT measurements by inputting the graphs into the software and requesting a simple analysis of the differences.

## Results

**Figure 1:**
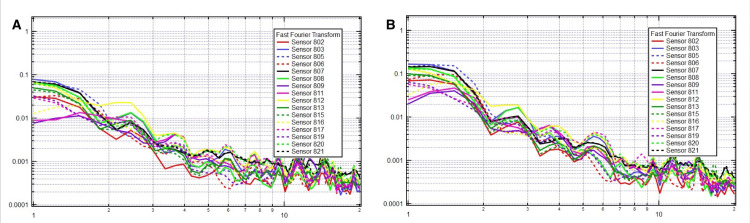
Sample patient electromagnetic frequencies before (A) and after (B) OMT. The x-axis is frequency in hertz on a logarithmic scale, and the y-axis is voltage on a logarithmic scale. OMT: osteopathic manipulative treatment

A total of 35 participants completed the study procedures, including EMF recordings before and after receiving suboccipital release OMT. There were 10 males and 25 females, all adults aged 18-65, with no contraindications to OMT and no history of prior surgery. The CV4 technique demonstrated an increase in synchronized brain activity in both the right and left hemispheres. This synchronization is evidenced by a higher number of brain areas firing simultaneously at the same frequency, resulting in less separation in amplitude and an overall increase in amplitude, with more pronounced peaks and valleys (Figure 1).

The effectiveness of OMT is indicated by increased synchronous firing and more prominent peaks, valleys, and amplitudes in the frequency range of 1 Hz to greater than 3 Hz. Specifically, when lower synchronized frequencies exceed 3 Hz up to 4 Hz, the remainder of the brain synchronously fires at multiple different frequencies.

**Figure 2:**
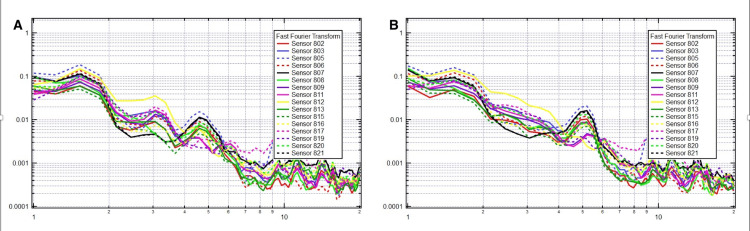
Sample patient showing flipping of frequency at 2.5 Hz from negative pre-OMT (A) to positive post-OMT (B). The x-axis is frequency in hertz on a logarithmic scale, and the y-axis is voltage on a logarithmic scale. OMT: osteopathic manipulative treatment

Effective OMT altered the frequency spectrum by flipping some frequencies, removing certain pre-OMT frequencies, and adding new ones (Figure 2). Although the specific frequencies subtracted and added are individualized to each patient, common patterns were observed, including the loss of frequencies in the range of 1.8 to 2.5 Hz and the gain of frequencies at 3 and 5 Hz. After treatment, there will be more loss of synchronicity and a lower amplitude of lower frequencies at 1.8, 2.5, and 3.0-3.5, particularly if negative, resulting in the synchronization and flattening of previously existing peaks and valleys. This loss of synchronicity and lower amplitude of pre-OMT frequencies primarily affects the left hemisphere of the brain. However, there will be a gain in frequencies and increased synchronicity and amplitude at the first derivatives of 1.3, 1.8 (negative), 2.5, 2.8, and 3.5, as well as bilaterally at frequencies of 3 and 5 Hz.

**Figure 3:**
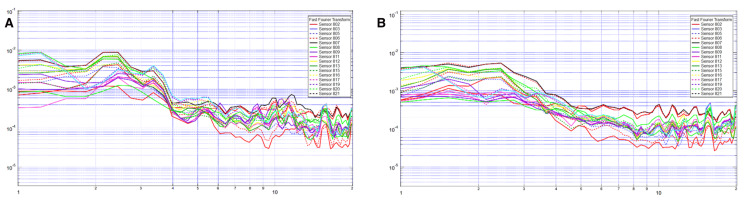
Sample patient showing differences between post-OMT (A) and post-meditation (B). The x-axis is frequency in hertz on a logarithmic scale, and the y-axis is voltage on a logarithmic scale. OMT: osteopathic manipulative treatment

Measurements collected after the two minutes of meditation show a smoothing of all peaks but a steeper valley at 4.5 Hz. It also brings the waves closer together and lowers the amplitude; this is especially true at 10 Hz (Figure 3).

Common changes observed post-OMT include an increased loss of synchronicity and lower amplitude in lower frequencies (1.8, 2.5, and 3.0-3.5 Hz), especially negative ones. Post-treatment, these peaks and valleys are synchronized and flattened. More pronounced loss of pre-OMT frequencies occurs predominantly in the left hemisphere. Additionally, there is an increased gain in synchronicity and higher amplitude at first derivatives of frequencies such as 1.3, -1.8, 2.5, 2.8, and 3.5 Hz, as well as an increased gain in synchronicity and higher amplitude at bilateral frequencies of 3 and 5 Hz. A summary of these changes is highlighted in Table 1.

**Table 1:** Summary of differences in EMF activity at baseline, after OMT, and after meditation. EMF: electromagnetic field; OMT: osteopathic manipulative treatment

Condition	Brain Activity (Synchronization)	Amplitude Changes	Frequency Changes	Hemispheric Impact
Baseline	Lower synchronization of brain activity	Varied amplitudes with less prominent peaks and valleys	Broad frequency range, with distinct separation	Affects both hemispheres, but no specific pattern noted
After OMT	Increased synchronization, more areas firing at the same frequency	Overall increase in amplitude, more pronounced peaks and valleys	Loss of lower frequencies (1.8-2.5 Hz), gain at 3 and 5 Hz	Increased synchronicity and amplitude, more left brain affected
After Meditation	Smoother brain waves with closer frequency synchronization	Lower amplitude, particularly at higher frequencies (e.g., 10 Hz), with a steeper valley at 4.5 Hz	Smoothing of peaks, fewer frequency separations	Impact observed bilaterally, with waves becoming closer together

## Discussion

The EMF helmet-induction sensor technology was able to detect differences in EMF activity following a single session of CV4. The changes were noted in EMF waveform patterns, including sharper morphology, slope stability, and intensified signaling at certain frequencies, indicating that the OMT technique facilitated cortical EMF dynamics. These findings demonstrate the potential utility of the helmet in quantifying the neurophysiological effects of OMT techniques.

Prior studies have identified overall decreases in variability in neural circuits in the post-injured state in swine and in individuals with neurologic conditions [6,7]. Following the OMT, it was noted that there was less variability in changes in slope. It is hypothesized that there is likely an appropriate balance of variability that may promote optimal neural activity and firing patterns, and these post-OMT changes may identify regression towards this homeostasis. Alternatively, as decreased variability of slope was identified in those with altered signaling, these changes in signaling patterns may represent early signal alterations resulting from modulation of activity prior to reaching a steady state. Future studies will be necessary to evaluate the long-term effects of neural potentiation and signaling following OMT.

The changes in variability in EMF waves after OMT suggest increases in synchrony and facilitation signaling between neuronal circuits. This implies modulation of neural communication, which was picked up by the helmet. The gentle mobilization involved in CV4 is thought to generate proprioceptive input that may modulate neural activity [17]. Normalization of aberrant signaling pathways could explain the improvements in electromagnetic waveform patterns following OMT. Through mechanical strain effects, OMT techniques can potentially activate specific cortical neurons while calming overactive areas, resulting in more organized EMF signaling. This aligns with prior research showing links between OMT and the autonomic system, providing a novel way to quantify these interactions [18-20]. Interestingly, in an animal study that evaluated the effects of EMF stimulation on neural signaling, it was found that the introduction of a targeted EMF signal promoted more synchronous activity immediately post-stimulation, and these swine exhibited improved histological recovery from the controlled cortical impact [7]. Although these findings in swine may not be entirely analogous, similar patterns with increased synchronicity and decreased variation in slope were observed immediately post-OMT, which are hypothesized to relate to the treatment effect.

In addition, a shift in frequencies was observed after successful OMT in patients with negative inflections at lower frequencies. This is similar to the swine model, which showed a flipping of the amplitude from negative to positive after stimulation with electromagnetic frequencies [7]. This study showed an improvement in histological changes after treatment of TBI induced in the pigs, so this similar flipping may indicate that OMT may have a similar result. OMT can be used as a noninvasive technique that requires no equipment to treat patients with brain injury. The more OMT is effective, the more there is synchronous firing and more peaks, valleys, and amplitudes between 1 and greater than 3 Hz. If greater than 3 Hz, the brain synchronously fires at many different frequencies.

The EMF changes following CV4 frequently occur in brain regions involved in emotional and cognitive processing. The left frontal lobe is thought to be particularly important for emotion regulation, as it contains key areas such as the dorsolateral prefrontal cortex and ventromedial prefrontal cortex, which are involved in the cognitive control of emotion and mood [21,22]. Enhanced electromagnetic signaling in the left frontal region following CV4 may indicate modulation of these pathways involved in emotional processing and stability. The helmet’s detection of EMF changes in the brain after OMT aligns with proposed effects on autonomic balance and affirms the treatment's influence on brain regions specialized for affective and self-regulatory functions. In addition, due to the placement of the sensors around the head, each signal wave generated corresponds to a specific area of the brain, further identifying a more precise location of dysfunction.

The frequencies that we can study in detail also correspond to delta, theta, and alpha waves. Multiple patients showed a decrease in higher frequency amplitudes, which can be related to decreased stress, anxiety, and hyperarousal. Patients also exhibit an increase in lower-frequency amplitudes, which can be linked to higher states of relaxation, meditation, and restoration.

To distinguish the effectiveness of OMT from simple meditation, as patients may also be in a state of relaxation, we compared the records after OMT and meditation. Meditation showed a difference by smoothing the waves and bringing all the waves together. Although this similarly indicates an increase in synchronization, the decrease in amplitude shows a different state of the brain waves than that of OMT. Thus, OMT not only puts a patient in a state of relaxation but also uniquely affects the brain, as measured by electromagnetic frequencies.

This proof-of-concept study provides evidence that the EMF helmet enables non-invasive, real-time measurements of OMT effects on cortical activity. The sensors provide an innovative tool for quantifying OMT outcomes using an objective neurophysiological biomarker. Future research can investigate the duration of EMF effects, their relationships to symptom improvements, and potential applications for treatment monitoring.

The limitations of this preliminary study include the small sample size from a single clinical site. Additional investigation is needed to reproduce the findings in larger, more diverse cohorts. Long-term monitoring with serial EMF recordings could provide insight into the lasting effects of OMT. Analyzing electromagnetic activity in other brain regions may also reveal additional networks modulated by OMT. In addition to suboccipital release, craniosacral therapy is another OMT that involves gentle stimulation of the cranial bones, sacrum, and fascia to induce hypothesized effects on the autonomic nervous system [23-28]. The pace and rhythm of cerebrospinal fluid may be influenced by craniosacral treatment, which is proposed to help regulate the balance between sympathetic and parasympathetic tone [29,30]. Therefore, like suboccipital release, craniosacral therapy may also modulate neural activity through connections with the autonomic system, meriting further research on associated neurophysiological effects.

## Conclusions

This study provides initial evidence that non-invasive EMF helmet-induction sensor technology (helmet) can identify changes in EMF activity following CV4 OMT. The induction sensors integrated into a lightweight, shielded helmet enabled quantitative analysis of cortical EMF dynamics before and after OMT. The changes detected in EMF waveform patterns, including intensified signaling, stabilization of variability in slope, and even a reversal of frequencies from negative to positive, indicate that OMT can produce effects that lead to neurological recovery.

The ability of the EMF helmet to discern OMT-related modifications in cortical EMF signaling demonstrates proof-of-concept for utilizing this tool to objectively evaluate neuromodulatory interventions. The helmet provides an innovative approach to quantify OMT outcomes based on neurophysiological biomarkers. Further research can help establish therapeutic mechanisms and guide OMT application through analyzing EMF changes associated with specific techniques. In summary, this preliminary study established the feasibility of using a non-invasive, lightweight helmet to capture EMF differences generated by cortical neurons following OMT. The findings showcase the effects of OMT on brain frequencies and the effectiveness of the helmet in monitoring these changes.
